# Diversity of immunization strongly impacts SARS-CoV-2 antibody function surrogates

**DOI:** 10.1038/s41541-025-01226-6

**Published:** 2025-07-29

**Authors:** Benoît Levast, Jérémie Becker, Carla Saade, Inès VuDuc, Kendra Reynaud, Camilo Broc, Charlotte Mignon, Natacha Mariano, Stéphanie Donnat, Shin-Yi Yu, Adrien Saliou, Viet-Dung Tran, Oxana Vratskikh, Céline Couturier, Stéphanie Geoffroy, Anely Tranchot, Christophe Vedrine, Tom Perisse, Lily Bruyere, Martin Killian, Bruno Pozzetto, COVID AuRA consortium, Dulce Alfaiate, Antonin Bal, Constance d’Aubarede, Vanessa Escuret, Jean-Baptiste Fassier, Nicolas Guibert, Amélie Massardier-Pilonchéry, Mary-Anne Trabaud, Philippe Vanhems, Mitra Saadatian-Elahi, Laetitia Henaff, Karen Louis, Laurent Beloeil, Cyril Guyard, Stéphane Paul, Arnaud Marchant, Sophie Trouillet-Assant

**Affiliations:** 1 BIOASTER, Lyon, France; 2 CIRI - Centre International de Recherche en Infectiologie, Univ Lyon, Université Claude Bernard Lyon1, Inserm, U1111, CNRS, UMR5308, ENS Lyon, Université Jean Monnet de Saint-Etienne, Lyon, France; 3 Department of Internal Medicine, CHU Saint-Etienne, Saint-Etienne, France; 4 Joint Research Unit Civils Hospices of Lyon-bioMérieux, Hospices Civils de Lyon, Pierre-Bénite, France; 5 INSERM, U1111, CNRS, UMR 5308, Université Jean Monnet, Immunology and Immuno-monitoring Laboratory, Saint-Etienne, France; 6 Department of Microbiology, CHU Saint-Etienne, Saint-Etienne, France; 7 European Plotkin Institute for Vaccinology, Université libre de Bruxelles, Bruxelles, Belgique; 8 Département des Maladies infectieuses et tropicales, Hôpital de la Croix-Rousse, Hôpital Edouard Herriot, Hospices Civils de Lyon, Lyon, France; 9 Institute of Infectious Agents, Hospices Civils de Lyon, Lyon, France; 10 Occupational Health and Medicine Department, Hospices Civils de Lyon, Université Claude Bernard Lyon 1, Université Gustave Eiffel, UMRESTTE, UMR T_9405, Lyon, France

**Keywords:** Vaccines, Applied immunology, Translational research, SARS-CoV-2

## Abstract

System serology offers a comprehensive approach to evaluate the humoral immune response by evaluating multiple parameters. In the present study, based on four groups of individuals with a different history of SARS-CoV-2 immunization, we analyzed the serum of 180 individuals based on six serological methods to better decipher their immunity. Through our analysis, against different SARS-CoV-2 antigens or variants, we report the importance of system serology to better decipher population immunity. Fc-dependent parameters are key factors underlying the variability of humoral immune response triggered by different schemes of SARS-CoV-2 immunization. With an evolving exposure to new variants, the acquisition of robust cross-reactive Fc-dependent effector functions are likely to be key to control viral replication when neutralizing antibodies are poorly cross-reactive. As booster vaccination remains a useful tool in periodically bolstering humoral immunity, particularly in vulnerable populations, studies should continue to evaluate the humoral immune response using system serology approach.

## Introduction

Throughout the coronavirus disease of 2019 (COVID-19) pandemic the main correlate of protection against severe respiratory syndrome coronavirus 2 (SARS-CoV-2) infection was the neutralization capacity of specific antibodies, mediated by the Fab region of the antibody^1–3^. Nevertheless, antibodies have effector functions that are mediated by the Fc region through binding to Fc receptors expressed by innate immune cells triggering Antibody-Dependent Cellular Cytotoxicity (ADCC), Antibody-Dependent Cellular Phagocytosis (ADCP), and Antibody-Dependent Complement Deposition (ADCD). These IgG effector functions are modulated by the structural diversity of IgG Fc determined by IgG1-4 subclasses and by their N-glycosylation profile^4–6^. Numerous studies report that Fc-effector functions can contribute to the control of infection, the protection against viruses, and viral clearance, notably in the context of respiratory syncytial virus (RSV) and influenza virus infections^7–9^. The role of Fc-effector functions has been widely covered by several groups during the pandemic^10–18^ and studies have demonstrated that different immunization schemes distinctly shape the immune response against SARS-CoV-2 when examining parameters individually^19–25^. Taken together, there is therefore a need for further studies to assess the overall impact of immunization schemes on the immune response.

System serology offers a comprehensive approach to evaluate the humoral immune response by evaluating multiple humoral parameters against different SARS-CoV-2 antigens such as the receptor binding domain (RBD) of the spike protein. In the present study, based on four groups of individuals with a different history of COVID-19 vaccination and SARS-CoV-2 infection, we investigated how different immunization schemes impact antibody neutralization and other effector functions using system serology for six different parameters. This has led to a more nuanced understanding of immune responses in COVID-19 context.

## Results

### Clinical characteristics of study population

**Fig. 1:**
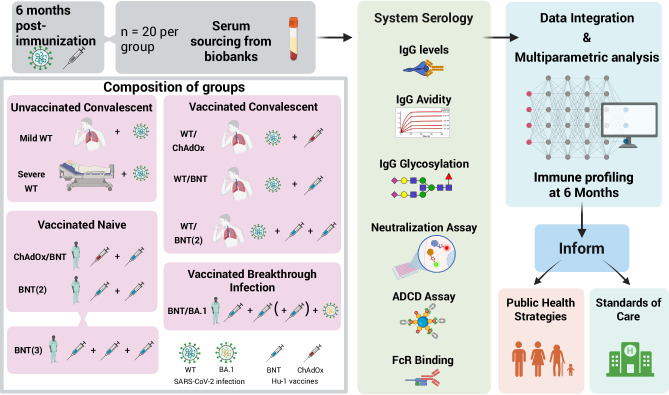
Study analysis workflow. Figure 1 illustrates the definition of the 9 groups of individuals (grey square) and the workflow of their samples after selection. A total of 180 individuals were selected from the 3 cohorts and subdivided into 9 different sub-groups according to immunization status, each composed of 20 participants: i) convalescent patients after severe (severe WT, *n* = 20) or mild (mild WT, n = 20) COVID-19 during the first wave of the pandemic; ii) vaccinated convalescent individuals (after mild COVID-19 during the first wave of the pandemic) who received either one (WT/BNT, *n* = 20) or two (WT/BNT (2), n = 20) doses of the Pfizer BNT162b2 mRNA vaccine or one dose of the adenoviral-based vaccine ChadOx1 (WT/ChAdOx, *n* = 20; this group is designed as “hybrid immunity”); iii) COVID-19 naïve individuals fully vaccinated with two (BNT (2), *n* = 20) or three (BNT (3), *n* = 20) doses of BNT162b2 or one dose of ChadOx1 followed by one dose of BNT162b2 (ChAdOx/BNT, *n* = 20), and iv) individuals vaccinated with two or three doses of the BNT162b2 vaccine followed by a breakthrough infection during the Omicron BA.1 wave (BNT/WT, *n* = 20). System serology (green central square) approach was to analyze 6 different technologies. Database was managed by BIOASTER to generate results, graph, ease scientific communication and potentially inform public health offices and standards of care. WT: Wild type (Lineage B) variant of SARS-CoV-2. IgG levels include anti-NTD and anti-RBD (WT, BA.1). Created in BioRender. Reynaud, K. (2025) https://BioRender.com/ak4i172.

A total of 180 individuals were selected from the three cohorts and subdivided into 9 different sub-groups according to immunization status, each composed of 20 participants: i) convalescent patients after severe (severe Hu-1, *n* = 20) or mild (mild Hu-1, *n* = 20) COVID-19 during the first wave of the pandemic; ii) vaccinated convalescent individuals (after mild COVID-19 during the first wave of the pandemic) who received either one (Hu-1/BNT, *n* = 20) or two (Hu-1/BNT (2), *n* = 20) doses of the Pfizer BNT162b2 mRNA vaccine or one dose of the adenoviral-based vaccine ChadOx1 (Hu-1/ChAdOx, *n* = 20; this group is designed as “hybrid immunity”); iii) COVID-19 naïve individuals fully vaccinated with two (BNT (2), *n* = 20) or three (BNT (3), *n* = 20) doses of BNT162b2 or one dose of ChadOx1 followed by one dose of BNT162b2 (ChAdOx/BNT, *n* = 20), and iv) individuals vaccinated with two or three doses of the BNT162b2 vaccine followed by a breakthrough infection during the Omicron BA.1 wave (BNT/BA.1, *n* = 20; Fig. 1). Of note, the latter group is the only one immunized with both the Hu-1 and BA.1 variants. All vaccines administered were monovalent vaccines targeting the Wuhan -Hu-1 S protein only. For each individual, the blood sample was collected at approximately 6 months post last immunization, i.e., 6 months post infection or last vaccine dose, except for the severe COVID-19 sub-group (Severe Hu-1) and vaccinated sub-group (Hu-1/ChAdOx), for which the median value was 9 and 7.2 months, respectively. The Severe Hu-1 group also distinguishes itself by a smaller proportion of females (50.0%) along with a higher median age (53 years) compared to the other groups. To prevent the immunization**-**to**-**sampling interval, age and sex from confounding the immunization effect, all system serology parameters were adjusted accordingly (see Methods section). Four samples were identified as outliers (abnormal FcγRIIIaV values) and were removed from subsequent analyses. A summary of the clinical and demographic data for each group is presented in Table 1.

**Table 1:** Patient characteristics

	Unvaccinated Convalescent		Vaccinated Convalescent			Vaccinated Naïve			BTI
	Mild WT	Severe WT	WT/ChAdOx	WT/BNT	WT/BNT (2)	ChAdOx/BNT	BNT (2)	BNT (3)	BNT/BA.1
**n**	20	20	20	20	20	20	20	20	20
**Female n (%)**	15 (75)	10 (50,0)	13 (65,0)	16 (80,0)	15 (75)	10 (50.0)	15 (75)	15 (75,0)	17 (80,0)
**Age at blood sampling (years). median (IQR)**	28,5 (25,0–33,5)	53,0 (42,5–65,8)	32,0 (26,5–35,3)	37,5 (32,5–40,3)	31,0 (26,0–38.75)	30,5 (28,8–32,3)	34,5 (30,8–39,3)	50,5 (37,3–60,0)	45,5 (28,0–54,3)
**Body mass index. median (IQR)**	22,4 (20,5–24,1)	26,2 (23,8–29,1)	23,4 (21,2–26,0)	23,6 (20,7-25,9)	22,3 (19,1–24,9)	22,5 (20,8–24,1)	24,2 (21,5–29,2)	24,1 (21,8–27,0)	22,3 (21,5–24,3)
**Presence of comorbidity,** * n (%)	6 (30,0)	NA	2 (10,0)	7 (35,0)	6 (30,0)	3 (15,0)	7 (35.0)	9 (45,0)	5 (20,0)
**Immunization to blood sample interval (days). median (IQR)**	191,5 (183,5–196,5)	292 (268,0–306,3)	218 (197,5–243,0)	185 (174,5–211,5)	224,5 (208,8–241.5)	183,5 (175,0–190,0)	190 (163,0–196,3)	184 (179,0-210,0)	190 (175,5–196,5)
**# of vaccinations**	NA	NA	1	1	2	2	2	3	2 (*n* = 7) 3 (*n* = 13)

^*^Recorded comorbidities include diabetes, chronic neurological condition, hypertension, rheumatism, hypothyroidism, cancer, chronic lung disease, cardiac condition, allergy, renal disease, liver disease, sickle cell disease.

*WT* Wuhan wild type variant, *ChAdOx* Chimpanzee Adenovirus Oxford nCoV-19 vaccine, *BNT* BioNTech 162b2, COVID-19 mRNA vaccine (Comirnaty^TM^), *BTI* (Vaccinated) Breakthrough Infection, *IQR* Inter-Quartile Range.

### System serology analysis discriminates against different immunization schemes

**Fig. 2:**
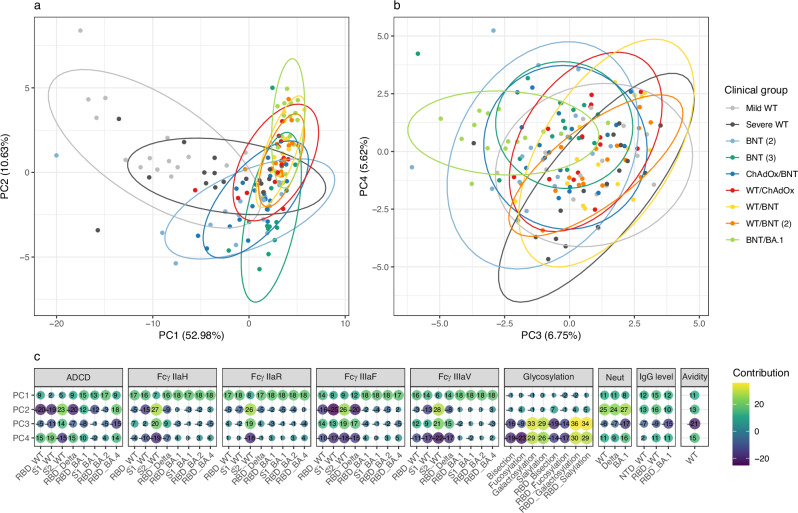
Principal component analysis on nine distinct clinical groups profiled with six system serology technologies. Sample projections onto PC1-2 (**a**) and PC3-4 (**b**), (**c**) heatmap of PCA loadings representing the contribution of each variable to each principal component.

Our first aim was to explore the potential discrimination of the nine different groups using immune profiling of six humoral parameters for a large range of SARS-CoV-2 antigens and variants (Fig. 1). A principal component analysis (PCA) was conducted; principal component one (PC1) explained 53% of the observed variance, PC2 11%, PC3 7%, and PC4 6%. The results show that mild WT (Hu-1) infected patients cluster away from the other groups along PC1 while BNT/BA.1 breakthrough patients can be differentiated along PC2. By contrast, PC3 and PC4 showed no clear association with known clinical or technical variables (Fig. 2b). Variable contributions to PC1 are more evenly distributed than in other PCs, reflecting that mild WT-infected individuals generally exhibit lower values across most parameters. While multiple features contribute to the variance captured by PC1, FcγR binding plays a notable role in distinguishing between conditions along this principal component, particularly FcγR binding to Omicron variants. For PC2, the main explanatory variables are attributed to ADCD activity and FcγR binding for pre-Omicron variants, as well as sero-neutralization for both pre-Omicron and Omicron variants. For PC3-4, Fc glycosylation, BLI, and to a lesser extent FcγR binding to pre-Omicron variants, exhibit the highest absolute loadings. Each principal component (PC1 to PC4) shows distinct patterns of contribution for individual parameters. For instance, anti-RBD IgG bisection and fucosylation contribute positively to PC4, whereas galactosylation and sialylation contribute negatively to it (Fig. 2c).

**Fig. 3:**
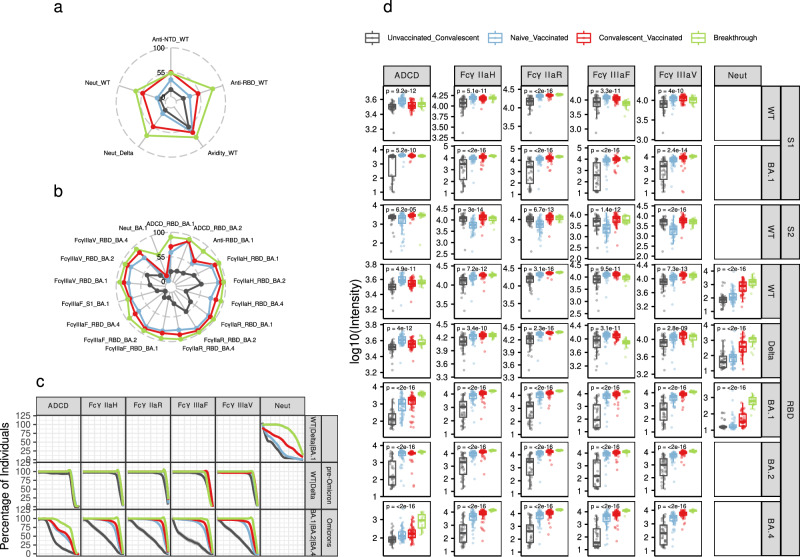
Analysis of the groups of individuals with different patterns of immunization. Radar plot showing the 20 features with highest variability (ANOVA), in early (**a**) and late (**b**) variants. Because ranges vary across platforms, the per-group medians are shown as percentage of the maximum value. **c** Breadth-potency curves representing the percentage of individuals with a response exceeding a given log_10_(intensity) level (ADCD activity, FcγR binding ability and sero-neutralization (Neut), see Methods section). **d** Boxplots of system serology activity (ADCD, FcγR binding ability and sero-neutralization against RBD, S1 and S2 subunits) measured in five variants across the four meta clinical groups.

Individuals were grouped into four meta-groups according to the different immunization schemes: i) “unvaccinated convalescent” (mild Hu-1 and severe Hu-1 patients), ii) “naive vaccinated” (BNT (2), BNT (3), and ChadOx/BNT vaccinated individuals), iii) “hybrid immunity” (Hu-1/ChadOx, Hu-1/BNT, and Hu-1/BNT (2) convalescent vaccinated individuals), and iv) “breakthrough infection” (breakthrough infection in vaccinated individuals, a different kind of hybrid immunity where the infection occurs after vaccination rather than before). Beyond its clinical relevance, this grouping is supported by the observation that most of the variation occurs across meta-groups, although notable differences persist between mild and severe unvaccinated convalescents across multiple parameters (Supplementary fig. 2). Additionally, we divided the antigens included for all parameters into two groups, early (pre-Omicron) and late variants (Omicrons), according to the time of circulation of each variant, Omicron BA.1 being the cut-off between the two groups. Thus, “pre-Omicron” variants refer to Hu-1(WT), 19 A and Delta, while “Omicrons” variants refer to BA.1, BA.2 and BA.4. Then, we aimed to evaluate how different immunization schemes shape antibody response against different variants. For both SARS-CoV-2 variants, the results show that individuals in the breakthrough infection group exhibit the highest levels of anti-RBD and anti-NTD antibodies, avidity, neutralization capacity as well as the strongest humoral response on most of the Fc-mediated parameters (FcγR binding and ADCD activity) compared to other groups (*p*-values < 0.05; Fig. 3a, b, Figure S3). Two exceptions can however be noted for responses to the pre-Omicron variants: breakthrough individuals exhibit a lower binding to FcγRIIIaF compared to hybrid immunity and naïve vaccinated individuals; naïve vaccinated individuals show the highest ADCD activity (Fig. 3c). By contrast, individuals in the unvaccinated convalescent group display a significantly lower magnitude of response for all Fc-dependent parameters against all RBD variants (*p*-values < 0.01; Fig. 3c). To assess whether antibody levels confounded the results, we repeated all analyses after adjusting for antibody levels, using WT and BA.1 strains for pre-Omicron and Omicron variants, respectively (Supplementary fig. 4). Key findings remained consistent: unvaccinated convalescents still exhibited overall reduced Fc-dependent functions across variants. However, after adjustment, in pre-Omicron variants the decreased FcγRIII and FcγRII binding in breakthrough infections became more pronounced, while differences in sero-neutralization between groups were attenuated, as evidenced by higher ANOVA p-values.

### Neutralization and FcγR binding activities correlate with a higher breadth coverage among the breakthrough population

We then aimed to estimate the breadth of coverage of antibodies in individuals from the four meta-groups. This measure reflects the capacity of antibodies to recognize and potentially counteract a diverse panel of variants, suggesting their broad protective efficacy against different viral strains. In pre-Omicron variants, although some differences - mainly between the unvaccinated convalescent group and the other three groups - were significant (Fig. 3c and Supplementary fig. 5), these differences were modest in magnitude. Individuals in the unvaccinated convalescent and breakthrough groups exhibited the lowest and highest values respectively, except for FcγRIIIaF (Fig. 3b, Supplementary fig. 5). Larger differences were observed for Omicron variants with breakthrough individuals still displaying the highest breadth scores, closely followed by convalescent and naïve vaccinated groups. Unvaccinated convalescents, on the other hand, exhibited much lower values. This result indicates that Fc-dependent parameters have a significantly higher breadth of coverage against Omicron variants after vaccination compared to a single Hu-1 infection. For neutralizing antibodies, greater variation is observed across groups with all pairwise comparisons being significant and a clear hierarchy emerging with unvaccinated convalescent individuals having the lowest magnitude of response and those in with a breakthrough infection having the highest (Supplementary fig. 5). Taken together, these results indicate that individuals in the breakthrough group exhibit the highest breadth of coverage for antibody Fc-dependent as well as Fab-dependent functions.

### The breakthrough population has a specific antibody Fc glycosylation profile

**Fig. 4:**
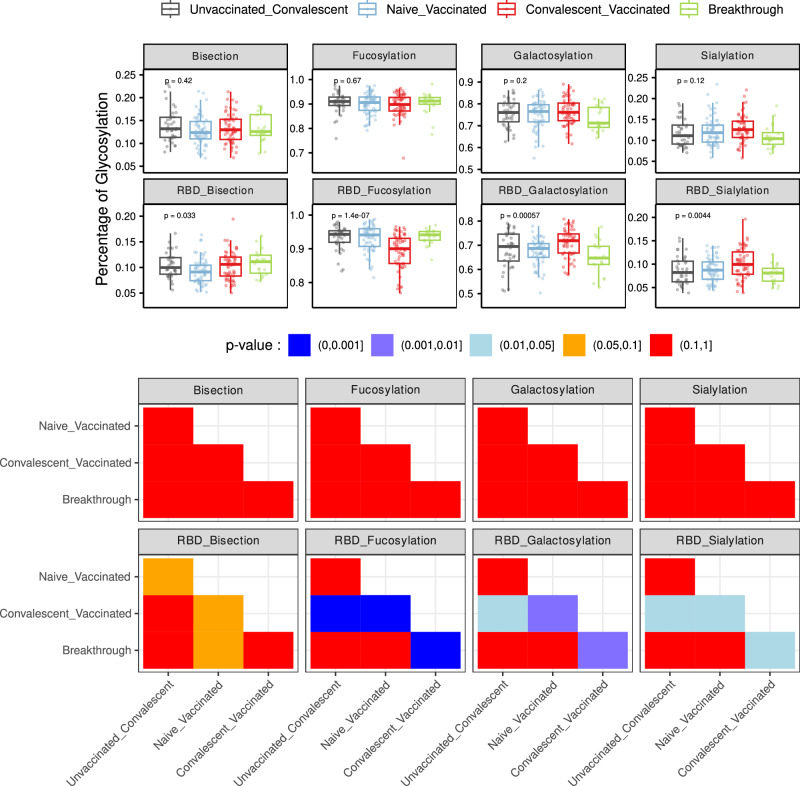
Boxplots of glycosylation (percentage of Bisection, Fucosylation, Galactosylation, Sialylation) in bulk (first row) and anti-RBD (second row) IgG1 measured in serum. Pairwise Student's t-test p-values across meta-groups, categorized into significance bins in bulk (third row) and anti-RBD (fourth row) IgG1.

To explore the potential role of antibody Fc biophysical characteristics, we investigated the impact of the different immunization schemes on antibody Fc glycosylation profiles. Proportions of individual glycans were analyzed for total IgG1 as well as SARS-CoV-2 Hu1 RBD-specific IgG Fc. Differences in proportions of galactosylation and sialylation were significant (ANOVA *p* < 0.05) at the level of both total and RBD-specific IgG1, the lowest proportions were detected in individuals with breakthrough infection and highest levels detected in the hybrid immunity group (Convalescent_Vaccinated, see pairwise Student's t-test in Fig. 4). Antigen specific differences in proportions of fucosylated IgG Fc were observed for RBD IgG Fc that were not observed at the level of total IgG1, with lowest proportions detected in the hybrid immunity group (Fig. 4).

## Discussion

Since 2019, the emergence of multiple SARS-CoV-2 variants has led to new waves of infection across the globe, with the RBD of SARS-CoV-2 spike protein being a key site for escape mutations^26^. Increasingly, studies demonstrated the role of Fc-dependent antibody effector functions in determining the outcome of SARS-CoV-2 infection, particularly in the absence of neutralizing antibodies against emerging COVID-19 variants^13,27^. In addition, murine mRNA SARS-CoV-2 vaccine studies indicated that neutralizing antibody responses induced by vaccination alone were not sufficient for protection against severe disease and that Fc receptor responses are essential for clinical protection against Omicron^11,13,16^. In addition, some studies have reported that a single exposure to a SARS-CoV-2, whether through infection or vaccination, does not induce a strong enough immune response to overcome the immune imprinting against the ancestral strain^28–30^. These studies also report that at least two exposures to a SARS-CoV-2 variants are needed to increase the proportion of antibodies specific or cross-reactive to that variant.

Consequently, in this study, we aim to decipher how different immunization schemes impact humoral response through a system serology approach including fifty-five (55) humoral parameters across eight pre-Omicron & Omicron antigens and six different methods. We demonstrated that in addition to the central role of antibody quantity and avidity illustrated herein, antibody Fc-dependent parameters are a key contributor to the variability of humoral immunity across nine groups of individuals with different histories of immunization against SARS-CoV-2. As previously reported, unvaccinated convalescent individuals had the lowest levels of SARS-CoV-2 specific humoral immune response across most studied parameters and breadth scores as compared to the other groups^31–34^.

SARS-CoV-2-naïve vaccinated individuals had higher levels of antibody Fc-dependent parameters across pre-Omicron and Omicron variants as compared to unvaccinated convalescent patients, but their neutralizing antibody levels were close in the two groups. Selva et al. observed that while a second dose of mRNA COVID-19 ancestral vaccine did induce stronger overall antibody-mediated FcγR engagement, it favored responses against the imprinted ancestral WT spike^35^. A recent study also showed that Omicron-specific antibodies made after receiving a single BA.5 bivalent vaccine were still largely cross-reactive to the ancestral spike^36^. As such, receiving monovalent variant-based vaccines could be more effective in promoting broader cross-reactive antibodies and overcome the limitations of immune imprinting by ancestral SARS-CoV-2 spike^37,38^. Nevertheless, the results of the present study indicate, for pre-Omicron antigens, that the breadth of antibody Fc-dependent parameters in unvaccinated convalescent patients were not markedly lower than that of individuals who had hybrid immunity. This suggests hybrid immunity could enlarge the repertoire of anti-viral immunity without increasing Fc functions.

Hybrid immunity has been commonly used to refer to cohorts of either COVID-19-convalescent vaccinees (recovered, then vaccinated) or individuals with breakthrough infections (vaccinated, then infected), despite the key determinant of such sequences in case of emerging epidemic^20,37,39^. Numerous studies have extensively documented hybrid immunity in COVID-19, highlighting the benefits of combining natural immunity and vaccination in various sequences^12,15,21–23,40–46^. Herein, we demonstrate that while both cohorts are conceptually similar, the order and sites of exposure (full virus in the respiratory tract versus spike antigen in the muscle), as well as their levels of preexisting immunity, has a significant impact on the levels of antibody Fc- and Fab-dependent parameters. Regarding Fab functions, individuals who experienced breakthrough infections had the highest neutralization capacity, anti-RBD IgG avidity, anti-RBD IgG levels, and breadth score compared to the other groups. This confirms results reported by studies that investigated these parameters individually^27,47,48^. In addition, we acknowledge that the characteristics and the breath of coverage are dependent on the variant responsible for the infection. Moreover, COVID-19-convalescent individuals who were subsequently vaccinated have been shown to develop broader cross-reactive antibody affinity maturation that can better engage Omicron subvariant BA.1 than vaccinated-only individuals^49,50^.

The highest levels of Fab- and Fc-dependent parameters across pre-Omicron and Omicron variants were observed in vaccinated individuals who had developed breakthrough infections. A recent study observed that variants with larger number of mutations, e.g., Omicron, induce lower FcγR functions compared to earlier variants of concern with fewer mutations^51^. Intriguingly, in this study reduced FcγR functions specifically to Omicron BA.2 as compared to BA.1 were also observed, suggesting that FcγR functions may be uniquely influenced by certain mutations. Bartsch et al. also reported that FcγR-functions against the full-VOC spike are less drastically impacted by mutations, likely reflecting the greater number of conserved epitopes harbored on the full-spike that can be targeted by both neutralizing and non-neutralizing antibodies. In contrast with the other Fc-dependent parameters, we observed that levels of antibodies binding to the low affinity FcγRIIIaF, and not the high affinity V variant, were lower in individuals with breakthrough infection as compared to vaccinated convalescents. This lower binding for the FcγRIIIaF was associated with higher levels of fucosylation of RBD-specific IgG1 in breakthrough individuals as compared to vaccinated convalescent individuals. The addition of a fucose to the conserved N-glycan at the asparagine 297 of IgG Fc is a key determinant of the affinity of IgG for the FcγRIIIa and for the activation of ADCC^27,52,53^. These results therefore suggest that individuals with breakthrough infection produce antibodies with higher potential for ADCC-dependent control of SARS-CoV-2. A new and important study also shows that the complement system can enhance SARS-CoV-2 neutralization titers for some vaccinated individuals and that this mechanism is likely mediated through the inhibition of viral attachment/entry to the host cell. Antibodies which bind outside the epitope for receptor binding may be dependent on the subsequent binding and deposition of complement proteins to then mask these epitopes^25^.

The present study has several potential limitations. First, we did not estimate the risk of infection due to the study design, thus a longitudinal study that follows the immune response throughout immunizations is needed to evaluate the risk of infection as well as describe the immune shift following repeated vaccination. The results also support the idea that mild COVID, versus severe COVID, is associated with reduced adaptive immunity in terms of quality and quantity, which emphasizes the need to specifically report disease severity for each infection event. Second, we did not evaluate the mucosal immune response and thus further studies, evaluating how different immunization schemes impact the overall profile of antibody function, need to be conducted. Third, unlike the other clinical groups, those in the breakthrough group were immunized with two variants (WT and BA.1) making comparisons challenging. Despite most of the antibodies in post-breakthrough infections being cross-reactive with antigens belonging to the WT ancestral strain, we acknowledge that some BA.1-specific antibodies were not analyzed specifically for each parameter, such as in the analysis of antibody glycosylation. Fourth, Fc-mediated effector functions are addressed by proxy measurements only (FcγR binding and glycosylation), and functional analysis, that can be impacted by FcγRIIIa-F/V variants, have not been assessed in this study. It is well described that the FcγRIIIa-F/V variants determine the affinity of FcγRIIIa binding to different IgG subclasses and correlate with effector function (FcγRIIIa-F/F homozygotes having lower antibody binding and ADCC activity compared to FcγRIIIa-F/V and V/V individuals). Also, the difference in FcγRIIa-H/R lies in the binding of IgG2, an IgG subclass that primarily targets glycans, and affects phagocytic activity. Therefore, functional analysis, taking into account the impact of FcγRIIIa-F/V variants, should be addressed in future studies to fully describe humoral response unlike similar systems serology studies done in other groups^54,55^. Fifth, the follow-up of circulating variants is a challenge with recent SARS-CoV-2 variants such as XBB.1.5 and JN.1 escaping neutralizing antibodies induced by previous vaccination and/or infection, and we decided to limit the analysis to a comparison between the antigen of the original Wuhan WT virus, early emerging variants (pre-Omicron) and the first emerging variants of concern following Omicron breakthrough. This also limits the risk of confounding immunization over a too long period as asymptomatic infections play a role in the long-term adaptive immunity^56^.

In conclusion, Fc-dependent parameters are key factors underlying the variability of humoral immune response triggered by different schemes of SARS-CoV-2 vaccination and infection. With an evolving exposure to new variants, the acquisition of robust cross-reactive Fc-dependent effector functions are likely to be key to the control of viral replication when neutralizing antibodies are poorly cross-reactive. As booster vaccination remains a useful tool in periodically bolstering humoral immunity, particularly in vulnerable populations, studies should continue to evaluate both neutralizing and non-neutralizing antibody responses.

## Methods

### Study design

We gathered 9 groups (and 4 meta-groups, see Fig. 1), each consisting of 20 randomly selected individuals, from 3 previously described cohorts. Convalescent patients with severe COVID-19 were selected from the NOSO-COR IMMUNO cohort (NCT04637867). Vaccinated individuals who experienced a breakthrough infection were drawn from the COVID-IVAC cohort (NCT05060939). COVID-19-naïve vaccinated individuals and convalescent patients after mild COVID-19 with or without vaccination were selected from the COVID-SER cohort (NCT04341142). Infected patients were considered to have a mild form of COVID-19 if they were symptomatic but did not require hospitalization. Hospitalized patients were considered to have a severe form of COVID-19 if their hospital stay was longer than 24 hours. Notable differences were observed across groups in terms of age, sex, and delay between immunization and blood sampling (Table 1). Consequently, all variables were adjusted before conducting the statistical analysis (see the Statistical analysis paragraph below). Written informed consent was obtained for all participants in all cohorts. Details for each clinical study are provided in the supplemental information section.

For the COVID-SER cohort, COVID-naïve health care workers (HCWs) infected with SARS-CoV-2, vaccinated against SARS-CoV-2, or both, were included in a prospective longitudinal cohort study conducted at the university hospital of Lyon (Hospices Civils de Lyon, Lyon, France). Blood sampling was performed approximately 6 months after the end of vaccination schedule or infection to collect and store blood cells and serum samples. Ethics approval was obtained from the national review board for biomedical research in April 2020 (ID-RCB 2020-A00932-37), and the study was registered on ClinicalTrials.gov: NCT04341142. The main inclusion criteria for this study were: seropositive HCWs screened for anti-SARS-CoV-2 Abs (Wantai Total Ab assay targeting the Wuhan RBD) or HCWs starting their COVID-19 vaccination schedule; at least 18 years of age; having given written informed consent and accepting a follow-up every 6 months.

For the NOSO-COR IMMUNO cohort, hospitalized patients were considered to have a severe form of COVID-19 if their hospital stay was longer than 24 hours. Blood sampling was performed approximately 6 months after SARS-CoV-2 infection. Ethics approval was obtained from the national review board for biomedical research in October 2020 (ID-RCB 20.02.27.69817), and the study was registered on ClinicalTrials.gov: NCT04637867. The main inclusion criteria for this study were: SARS-CoV-2 confirmed by reverse transcription polymerase chain reaction (RT-PCR); at least 18 years of age; having given written informed consent and accepting a follow-up of 6 months.

For the COVID-IVAC cohort, a prospective test-negative study was conducted at the university hospital of Lyon (Hospices Civils de Lyon, Lyon, France). Clinical, microbiological, and serological data were collected for all included individuals. Blood sampling was performed approximately 6 months after Omicron BA.1 breakthrough infection to collect and store serum samples. The COVID-IVAC study was registered on ClinicalTrials.gov: NCT05060939. Ethics approval was obtained from the national review board for biomedical research in July 2021 (ID-RCB 2021-A01877-34). The main inclusion criteria of the study were: at least 18 years of age; having given written informed consent and accepting a follow-up of 6 months.

We identified the occurrence of breakthrough infections in these cohorts by assessing RBD IgG titers using the bioMerieux VIDAS SARS-CoV-2 IgG II (9COG) diagnosis kits (bioMérieux, 424114). In our hands, this assay was more reliable than monitoring seroconversion against the N antigen^57^. A breakthrough infection was identified by a rebound of RBD Ab titers instead of the steady decline that normally occurs in the absence of viral exposure. This follow-up of the RBD Ab titers revealed that none of the participants became infected/reinfected in the 6 months between vaccination/infection and blood sampling.

### Quantification of anti-RBD and anti-NTD IgG

Recent studies have demonstrated that repeated vaccination against the ancestral strains leads to an immune imprinting that is often not overcome by a single infection or vaccination against a VOC^28–30^. Indeed, these studies report that after a single exposure to a SARS-CoV-2 VOC, the majority of antibodies induced are either specific or cross-reactive to the ancestral strain. Therefore, to simplify the present study, we only evaluated the levels of IgG-specific to the receptor binding domain (RBD) of the ancestral and BA.1 strains. Wuhan Hu-1(WT) RBD-specific IgG were measured using VIDAS SARS-CoV-2 IgG II (9COG) diagnosis kits (bioMérieux, 424114), according to the manufacturer’s recommendations. For standardization of these assays to the first WHO international standard, the concentrations were transformed to Binding Antibody Unit (BAU)/mL using the conversion factors provided by the manufacturer. In addition, a custom SARS-CoV-2 multiplex assay was designed to quantify the amount of IgG targeted the BA.1.1 RBD (Sino Biological, 40592-V08H129) and NTD (“in house”, courtesy of Timothée Bruel, Pasteur Institute, Paris) antigens. As positive control of the assay, Tetanus toxoid (Sigma Aldrich, 582231) and influenza hemagglutinin H1Cal2009 (Sino Biological, 11085-V08H) were also added, while BSA-blocked beads were included as negative controls. The antigens were covalently coupled to Bio-Plex Pro Magnetic COOH beads (Bio-Rad) using a two-step carbodiimide reaction as per manufacturer’s instructions, in a ratio of 10 million beads-to-100 μg of antigen. The antigen-coupled beads were resuspended in a storage buffer (PBS, 0.05% sodium azide) as one million beads per 100 μL, and stored in the dark at 4 °C before use. Results were expressed in Mean Fluorescence Intensity as previously described^58^.

### Live Virus Neutralization

A 50% microneutralization assay was used for the detection and titration of neutralizing Abs, as previously described^19^. A 10-fold dilution of each serum specimen in Dulbecco’s Modified Eagle’s Medium (DMEM) high glucose (Sigma-Aldrich, D6429) culture medium was first heated for 30 min at 56 °C to avoid complement-related reduction of the viral activity. Two percent of heat-inactivated Fetal Bovine Serum (FBS; Eurobio scientific, CVFSF06-01) and 1% of penicillin (10,000 UI/mL) and streptomycin (10,000 UI/mL; Eurobio scientific, CABPES01-0U) were added to the culture medium. Serial 2-fold dilutions (tested in duplicate) of the serum specimens in supplemented culture medium were mixed with live SARS-CoV-2 virus strains (19 A, Delta, BA.1). After gentle shaking and a contact of 30 minutes at room temperature in plastic microplates, 150 μL of the mix was transferred into 96-well microplates covered with Vero E6 cells (ATCC CRL-1586, not authenticated but regularly tested for mycoplasma contamination). The plates were incubated at 37 °C in a 5% CO_2_ atmosphere. Infection-induced cytotoxicity was evaluated by microscopy 5 days later and neutralization was recorded if more than 50% of the cells present in the well were preserved. All experiments were performed in a biosafety level 3 laboratory. The different viral strains that were used were sequenced and deposited on GISAID [GISAID accession numbers: EPI_ISL_1707038, 19 A (B.38); EPI_ISL_1904989, Delta (B.1.617.2); and EPI_ISL_7608613, Omicron BA.1 (B.1.1.529)].

### Bio-Layer Interferometry (BLI) Assay

Avidity of Wuhan Hu-1 RBD-specific IgG from serum was based on off-rate constant measurement (k_off,_ s^−^^1^, as the binding kinetic of the antigen-antibody complex), which is not impacted by antibody concentration, as previously described^19^. Total IgG were purified from 100 µL of each serum sample using Magne Protein G Beads (Promega, G7471) according to the manufacturer’s instructions. BLI assays were performed on purified IgG with an Octet RED96 instrument (Sartorius) using Octet amine-reactive biosensors (AR2G; Sartorius, 18-5092). Kinetics of BLI were carried out at 30°C using standard kinetics acquisition rate settings (5.0 Hz, 1000 rpm). Briefly, after hydration in water, AR2G sensors were activated for 10 minutes in 20 mM 1-Ethyl-3-[3- dimethylaminopropyl]-carbodiimide hydrochloride (EDC; Sartorius, 18-5095) and 10 mM Normal Human Serum (NHS) before antigen loading. SARS-CoV-2 (2019-nCoV) Spike RBD-His Recombinant Protein (SinoBiological, 40592-V08B) 10 µg/mL in 10 mM sodium acetate pH6 was amine coupled to AR2G sensors for 10 min using octet amine-coupling reagents (Sartorius, 18-5095). Then, the sensor surface was inactivated with a solution of 1 M ethanolamine pH8.5. RBD-loaded AR2G sensors were dipped in 1X PBS (pH7.4; Thermo-Fisher, A1286301) for 2 min to establish a baseline time course before a 10 min association in purified serum IgG. Purified IgG were assayed from undiluted or diluted samples (only one dilution per sample, range from 1:2 to 1:50 in PBS), according to RBD-specific IgG titers of each sample. The dissociation step was monitored for 10 min by dipping sensors in 1X PBS into the wells used to collect the baseline. To validate each run, a positive inter-plate control was monitored. Samples with no significant binding were not considered for analysis. Dissociation rates were determined using Data Analysis HT 11.1 software (Sartorius), according to a 1:1 binding model.

### Fcγ receptors (FcγR) binding assay

Experiments were performed to analyze the binding of antigen-specific IgG to the R and H variants of Fc**γ**RIIa, a key receptor involved in IgG-dependent phagocytosis, and to the F and V variants of the Fc**γ**RIIIa, the receptor triggering antibody-dependent NK cell activation. All parameters were measured using a high throughput multiplex method using fluorescent microspheres coated with antigens.

The capacity of antigen-specific antibodies to bind FcγR was analyzed using a 96-well-based multiplexed Luminex assay (Bio-Rad, 171025001). Serum samples were diluted 1:250, 1:2500 and 1:25000 in PBS (pH7.4; Thermo-Fisher, A1286301), and incubated with antigen-coated beads for 2 hours with agitation at room temperature. After washing steps, the beads were incubated with biotinylated FcgR coupled to Streptavidin-R-Phycoerythrin (Agilent, PJ31S) for 1 hour with agitation at room temperature. For these experiments 2 allelic variants of the FcγRIIa, FcγRIIaR (Forlab, CDA-H82E5) and FcγRIIaH (Forlab, CDA-H82E6), as well as 2 allelic variants of the FcγRIIIa, FcγRIIIaF (Forlab, CDA-H82E8) and FcγRIIIaV (Forlab, CDA-H82E9), were used. IgG were then incubated with antigen-coated beads (for 2 h with agitation at room temperature). Samples were then incubated with human serum (Sigma-Aldrich, S1764) at a dilution of 1:50 at 37 °C for 30 min. A biotinylated monoclonal anti-human C3d antibody (Quidel, A207) was added for 30 min at room temperature. Finally, Streptavidin-RPE (Agilent, PJ31S) was added to each well and incubated at room temperature in the dark for 30 min.

### Antibody Dependent Complement Deposition (ADCD) assay

Experiments were performed to analyze the deposition of complement by antigen-specific IgG. All parameters were measured using a high throughput multiplex method using fluorescent microspheres coated with antigens.

Complement deposition was measured on a Bio-Plex-200 array reader (Bio-Rad, 171000201) and data were expressed as mean fluorescence intensity. Assays performed without IgG and with heat-inactivated human serum (Sigma-Aldrich, S1764) were used to determine the background signal. Serial dilution of a pool of serum collected from individuals with COVID-19 hybrid immunity (natural infection followed by vaccination) were used as a standard curve. The antigens used for this experiment were Wuhan Hu-1 S1 (40591-V08H), Wuhan Hu-1 S2 (40590-V08H1), Omicron BA.1 S1 (40591-V08H41), 19 A RBD (40592-V08H), Delta RBD (40592-V08H91), BA.1 RBD (40592-V08H129), BA.2 RBD (40592-V08H123) and BA.4 RBD (40592-V08H130), all purchased from Sino Biological.

### Antibody glycosylation analysis

#### Purification of bulk IgG

Serum and plasma samples were randomized (computation algorithm) to avoid experimental bias prior to starting sample preparation. Bulk IgG were isolated from serum or plasma by protein G, according to the supplier’s procedure (MultiTrap; Cytiva, 28-9031-35). Before immunoprecipitation of anti-RBD IgG from bulk IgG, bulk IgG were desalted using Zeba Spin Desalting Plate (Thermo-Fischer Scientific, 89807). Bulk IgG were quantified using a spectrophotometer (Thermo-Fischer Scientific, NanoDrop) at A280 nm.

### Immunoprecipitation of anti-RBD-specific IgGs from bulk IgGs

N-hydroxysuccinimide sepharose medium (NHS) was used to coat RBD protein (SARS-CoV-2 [2019-nCoV] Spike RBD; SinoBiological, 40592-V08B) and purify RBD-specific IgG from total IgG. For RBD immobilization, 20 µg of RBD protein were coated to 50 µL NHS according to the manufacturer’s protocol (Cytiva). In brief, NHS medium was activated by ice cold 1 mM HCl. On the other hand, RBD protein was added in the coupling buffer (0.15 M Triethanolamine, 0.5 M NaCl). Then, RBD protein was mixed with NHS at 50 rpm, for 4 hours at room temperature. After coupling, the reaction was quenched by 2 blocking buffers (0.5 M ethanolamine, 0.5 M NaCl and 0.1 M sodium acetate, 0.5 M NaCl). The anti RBD-IgG were eluted by 100 mM Glycine-HCl, pH2.5, and followed by neutralization by 1 M Tris-base buffer at pH10.

### Protein digestion and cleanup

Protein samples were adjusted with pure water to obtain 20 µg in 25 µL. Lysis and digestion were performed with iST high-throughput preparation kit (PreOmics, P.O.00165), following the manufacturer’s instructions. Peptide samples were then dissolved in 0.1% formic acid to 0.25 µg/µL (for bulk IgG analysis) and 0.05 µg/µL (for anti-RBD IgG analysis) for analysis on QExactive and Exploris 480, respectively. Batches and quality control pool were thawed at the same time and conditions before analysis.

### Bulk IgG glycopeptide analysis

Bulk IgG glycopeptide analysis was performed on an Ultimate 3000 RSLC Nano system coupled to a QExactive mass spectrometer (Thermo-Fisher Scientific). The protein digest (0.25 µg, 1 µL) was loaded onto a 15 cm EasySpray PepMap™Neo column (75 µm internal diameter, particle size 2 µm; Thermo-Fisher Scientific, ES75150PN), maintained at 45 °C, and separated at a flow rate of 300 nL/minute using a gradient of 2.5% to 43.8% of solvent (0.1% formic acid in 80% acetonitrile) in 45 minutes. Total run time, including column wash and re-equilibration, was 60 min. Top 12 data-dependent acquisition (DDA) was applied.

### Anti-RBD IgG glycopeptide analysis

Anti-RBD IgG glycopeptide analysis was performed on a Vanquish Neo system (Thermo-Fisher Scientific) coupled to an Exploris 480 mass spectrometer (Thermo-Fisher Scientific). The protein digest (0.25 µg, 5 µL) was loaded in a 5 mm Pepmap column (0.3 mm internal diameter, particle size 5 µm; Thermo-Fisher Scientific, 11362223) at a flow rate of 60 µL/minute and maintained at 60 °C in a 0.1% formic acid solvent. Then, peptides were separated in a 50 cm EasySpray PepMap™Neo column (75 µm internal diameter, particle size 2 µm) at a flow rate of 300 nL/minute and maintained at 60 °C. Solvent was 0.1% formic acid in 80% acetonitrile.

### Glycopeptide identification

The Mass spectrometry (MS) and MS/MS data were first evaluated by Preview (Protein Metrics Inc. Version 5.2.5) and Byonic (Protein Metrics Inc. Version 5.2.20). The general parameters were protein sequences of Fc domain of IgG1 (Uniprot ID, P01857), IgG2 (Uniprot ID, P01859), IgG3 (Uniprot ID, P01860) and IgG4 (Uniprot ID, P01861) with semi specific digestion at R and K residues, allowing up to 2 missed cleavages, with the precursor ion mass tolerance set at 10 ppm and the fragment ion mass tolerance at 20 ppm. The built-in N-glycan library of “182 human no multiple fucose” was applied.

### Glycopeptide quantification

Open-source Skyline software (version 22.2; MacCoss Lab, University of Washington) was used for quantification of glycosylated peptides by using DDA with MS1 filtering function. The imported peptide sequences for quantification were Fc-glycosylated peptides of IgG1, TKPREEQYNSTYR; IgG2, TKPREEQFNSTFR; IgG3, TKPREEQYNSTFR; and IgG4, TKPREEQFNSTYR. The relative abundance of each glycopeptide was calculated by normalizing its peak area to the sum of peak areas of all targeted glycopeptides in each sample.

### Statistical analysis

All parameters were log_10_ transformed (except glycosylation that is expressed as percentage) and adjusted for sex, age and delay effects, the latter being defined as the number of days between last immunization and blood sampling. By contrast, the number of immunizations was not adjusted for in the analysis, as it provided no additional information beyond what was already captured by the clinical groups, as demonstrated by the likelihood ratio test (Figure S1). A PCA was conducted to identify the main sources of variation.

Breadth-potency curves were generated by computing the proportion of viral variants that elicited an antibody response exceeding a threshold, evaluated across the range of log10(intensity) response (ADCD activity, FcγR binding ability and sero-neutralization). Similarly, breadth scores, which summarize the overall potency of an antibody response across viral variants, were calculated as the geometric mean of response log₁₀(intensity) across all tested variants. The score was computed in each individual and dilution for ADCD, FcγR, and sero-neutralization. The optimal dilution was subsequently selected as having the largest area under the Breadth-potency curve (AUC). Since clinical groups were sampled across different COVID-19 waves, breadth-potency curves were stratified into early (19 A, Delta) and late (Omicron BA.1, BA.2, BA.4) variants. Mean comparisons were conducted using both Welch’s t-test (two groups) and ANOVA (all groups).

## Supplementary information

Supplementary Figures

## Data Availability

The datasets generated and/or analyzed during the current study are subject to controlled access due to private funding by BioMérieux, Sanofi and Boehringer Ingelheim. The datasets are confidential due to privacy issues but are available from the corresponding author on reasonable request. Response to requests is expected in a timeframe of one month and data use agreement may be requested.
