# Supplementary material for: Diversity of immunization strongly impacts SARS-CoV-2 antibody function surrogates
Source: NPJ Vaccines. 2025 Jul 29;10:175. doi: 10.1038/s41541-025-01226-6 (PMC12307949; doi:10.1038/s41541-025-01226-6)
Supplement: Supplementary file 1 — Supplementary Figures [file 41541_2025_1226_MOESM1_ESM.pdf]

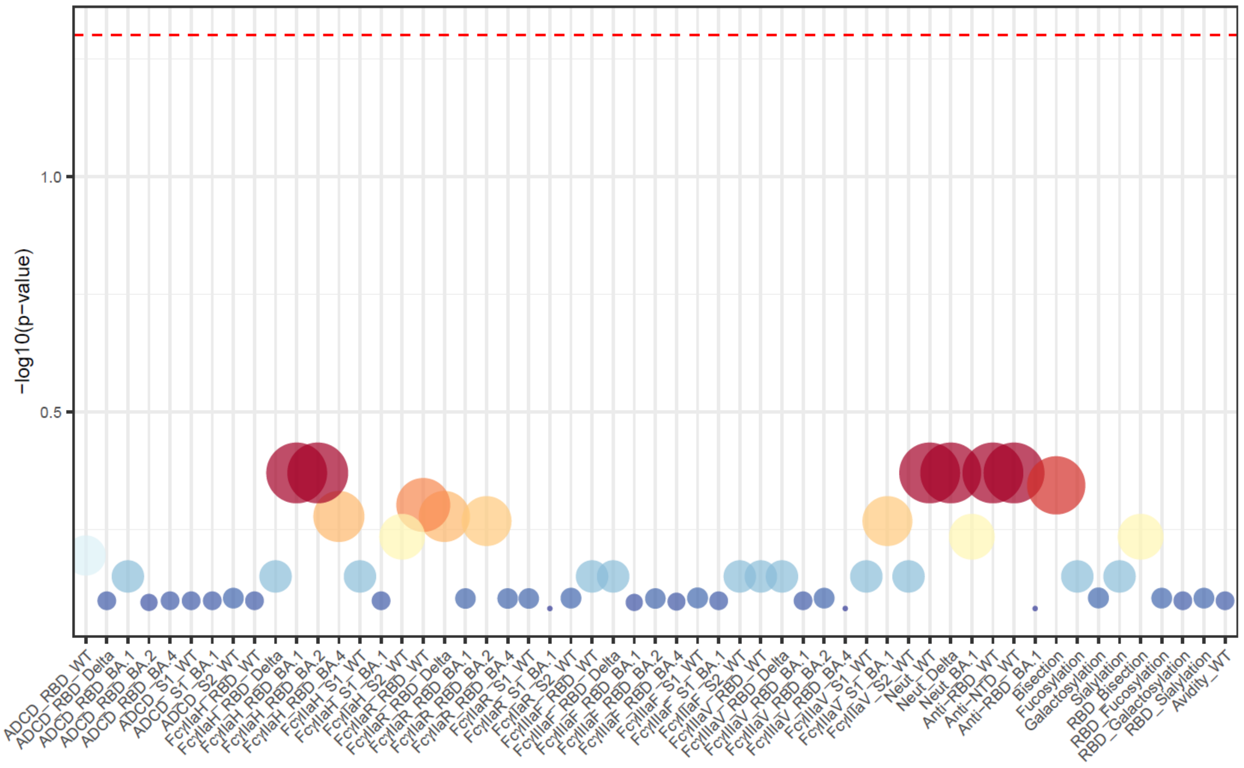

Supplementary Figure 1: Likelihood ratio test results evaluating the effect of the number of immunizations on all parameters. Two linear models (restricted and full) were fitted for each parameter. Both models included the effects of sex, age, delay, and meta-group as confounders. The full model additionally incorporated the number of immunizations. The dashed red line indicates the significance threshold (p-value = 0.05). The y-axis shows -log10(p-values).

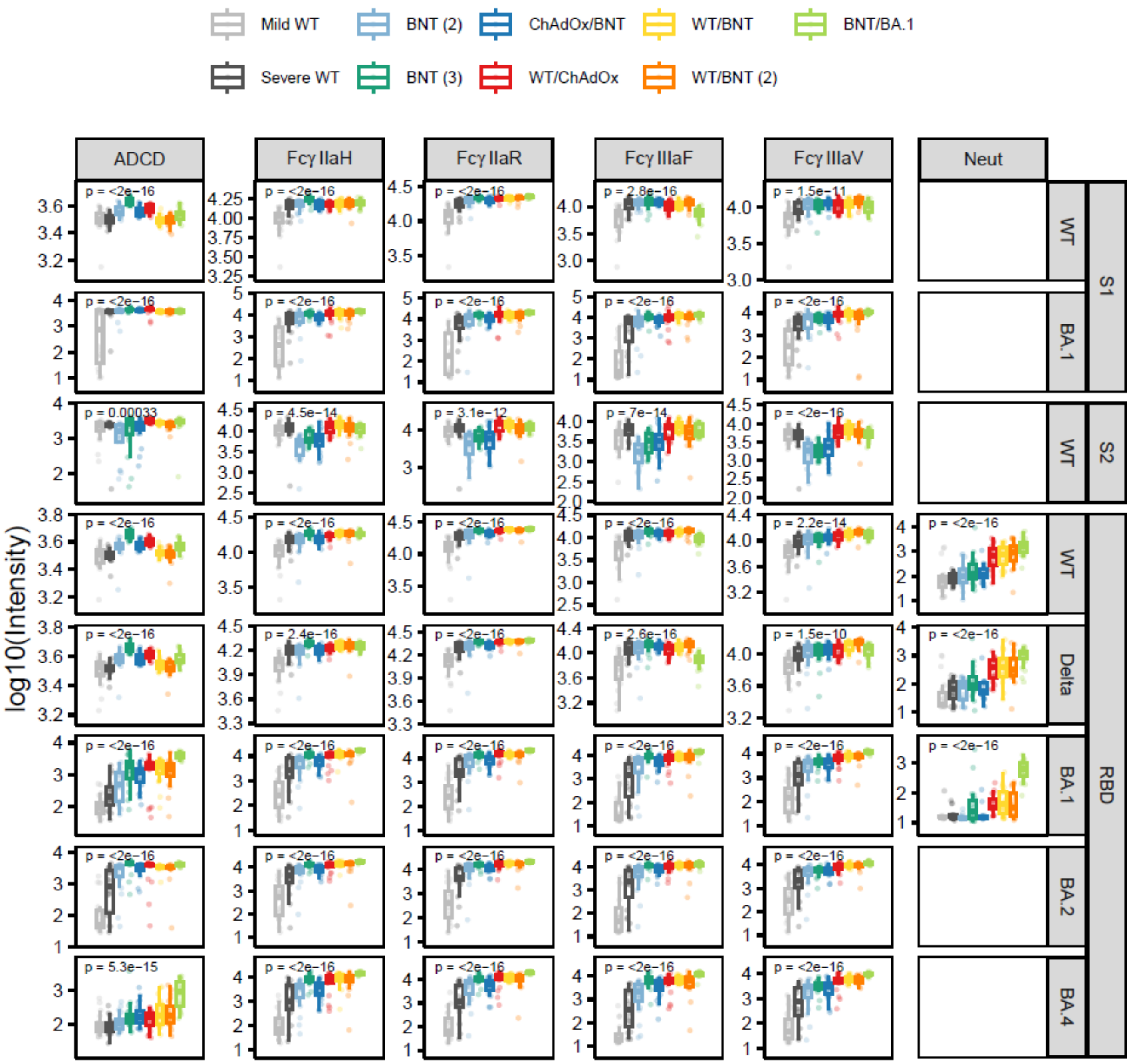

Supplementary Figure 2: Boxplots of system serology activity (ADCD, FcγR binding ability and sero-neutralization against RBD, S1 and S2 subunits) measured in five variants across the nine clinical groups.

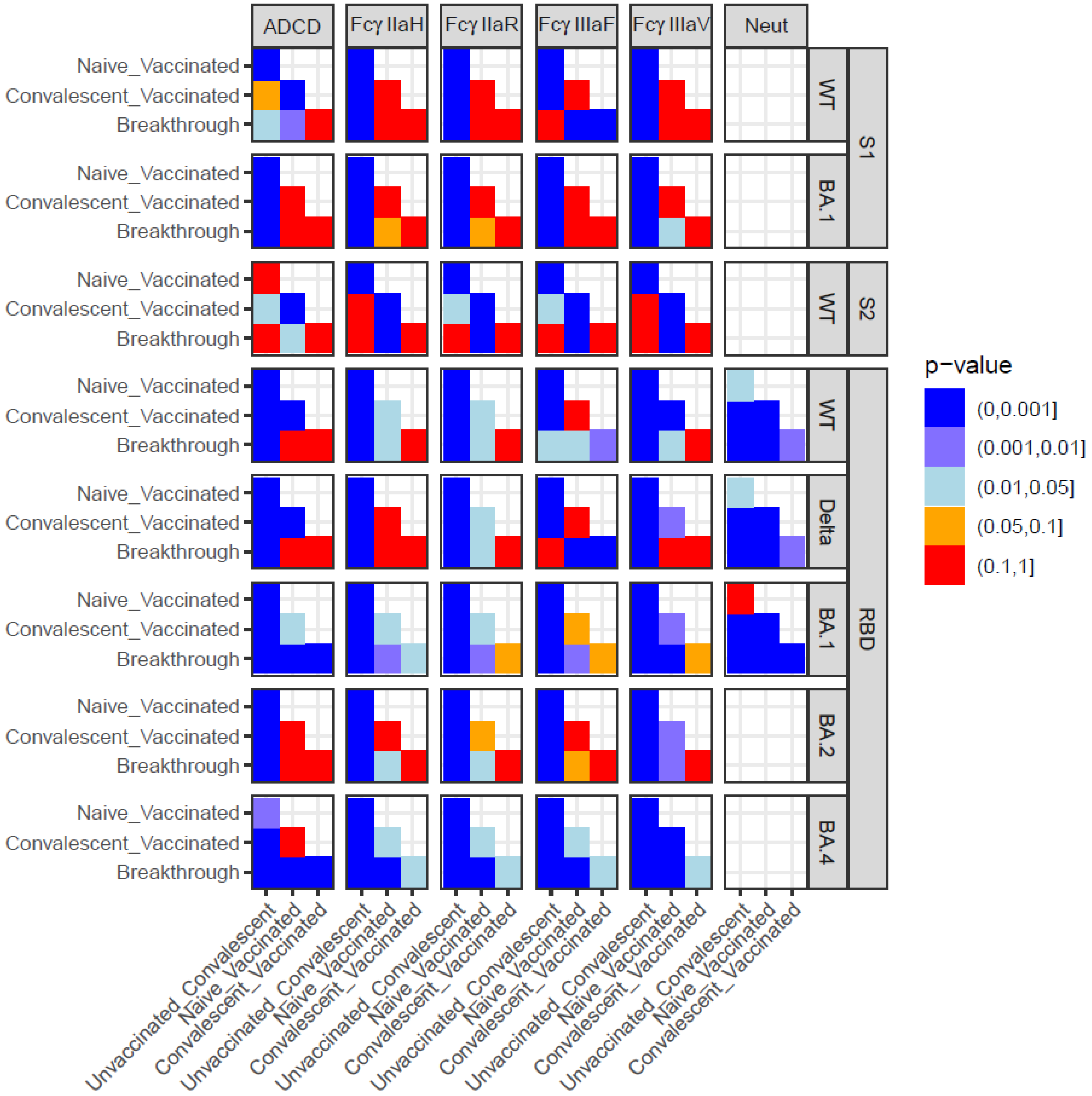

Supplementary Figure 3: Pairwise t-test p-values across meta-groups are shown across four meta-groups in five variants, categorized into five significance bins.

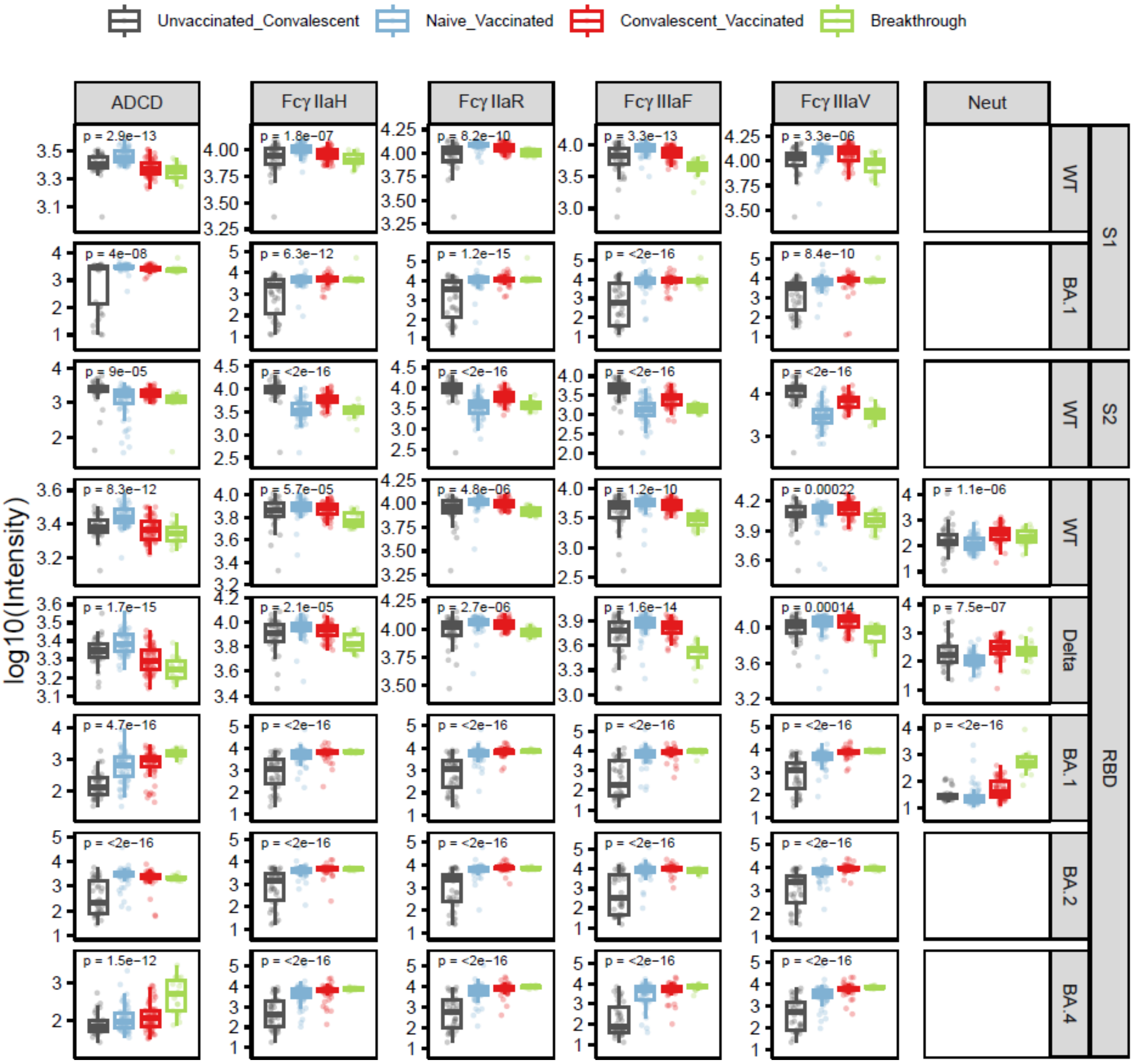

Supplementary Figure 4: Boxplots of system serology activity (ADCD, FcγR binding ability and sero-neutralization against RBD, S1 and S2 subunits) measured in five variants across the four meta groups after adjusting for antibody level (for comparison before adjustment, see Figure 3d).

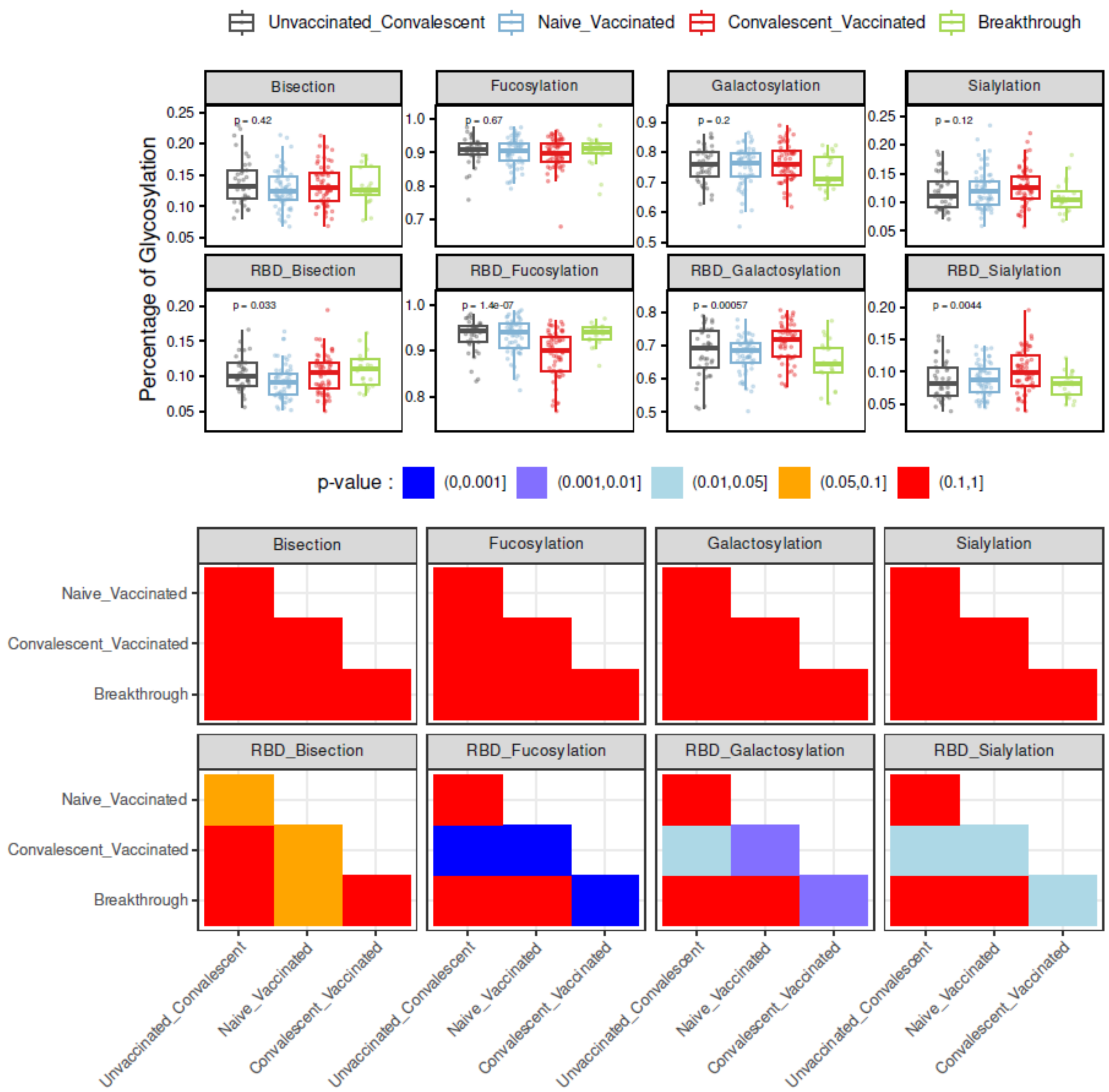

Supplementary Figure 5: (a) Boxplots of breadth score computed across three sets of variants on ADCD, FcR binding and sero-neutralization parameters. (b) Pairwise t-test p-values across meta-groups are shown, categorized into five significance bins.
